# Soil natural capital in europe; a framework for state and change assessment

**DOI:** 10.1038/s41598-017-06819-3

**Published:** 2017-07-27

**Authors:** David A. Robinson, Panos Panagos, Pasquale Borrelli, Arwyn Jones, Luca Montanarella, Andrew Tye, Carl G. Obst

**Affiliations:** 1 NERC–Centre for Ecology and Hydrology, Deiniol Rd, Bangor, LL57 2UW United Kingdom; 2 European Commission, Joint Research Centre, Via E. Fermi 2749, Ispra (VA), I-21027 Italy; 3 NERC–British Geological Survey, Environmental Science Centre, Keyworth, Nottingham, NG12 5GG United Kingdom; 4 Institute for the Development of Environmental-Economic Accounting (IDEEA) & Melbourne Sustainable Society Institute, University of Melbourne, Parkville, Vic Australia

## Abstract

Soils underpin our existence through food production and represent the largest terrestrial carbon store. Understanding soil state-and-change in response to climate and land use change is a major challenge. Our aim is to bridge the science-policy interface by developing a natural capital accounting structure for soil, for example, attempting a mass balance between soil erosion and production, which indicates that barren land, and woody crop areas are most vulnerable to potential soil loss. We test out our approach using earth observation, modelling and ground based sample data from the European Union’s Land Use/Cover Area frame statistical Survey (LUCAS) soil monitoring program. Using land cover change data for 2000–2012 we are able to identify land covers susceptible to change, and the soil resources most at risk. Tree covered soils are associated with the highest carbon stocks, and are on the increase, while areas of arable crops are declining, but artificial surfaces are increasing. The framework developed offers a substantial step forward, demonstrating the development of biophysical soil accounts that can be used in wider socio-economic and policy assessment; initiating the development of an integrated soil monitoring approach called for by the United Nations Intergovernmental Technical Panel on Soils.

## Introduction

Soils underpin our very existence through food, feed, fibre and timber production, as well as through earth system functions that support the delivery of other ‘ecosystem services’^1, 2^. It is critical that we govern our soil resource^3^ so it provides multiple soil functions that support the delivery of ecosystem services in order to meet the United Nations Sustainable Development Goals (SDG’s)^4^, and not simply pursue economic gain. The Sustainable Development Goals continue a shift in thinking regarding global policy, from the early focus on economic sustainability to the three pillars of sustainable development, economic, social and environmental^5^. Recent work shows conceptually how soil resources underpin soil function, the delivery of ecosystem services and the SDG’s^4^. The current gap is a monitoring and assessment information system that can inform policy, regarding progress on achieving economic, social and environmental goals.

The United Nations System of Environmental and Economic Accounting (SEEA)^6^ is well positioned to provide an information system that can support this effort. SEEA offers a broad-scale monitoring tool that is gaining global momentum. In the European Union the development of European accounting is one of Eurostat’s challenges, although they are to date focusing on environmental flows and not resources. Environmental accounts come under regulation (EU) 691/2011, which provides, ‘the legal framework for a harmonised collection of comparable data from all EU Member States and the European Free Trade Association (EFTA) countries’^7^; the EU accounts are consistent with SEEA. With regard to soils, Obst^8^ recently commented in Nature that, ‘Integrating information on soil resources with other measures of natural capital and economic activity remains one of the least developed areas of the United Nations SEEA.’ Therefore, there is a need to develop an appropriate framework for soil reporting and data integration starting with the SEEA Central Framework (SEEA-CF), focused on extent and mass, then SEEA experimental ecosystem accounts^9^ (SEEA-EEA) which are being developed that will require more emphasis on condition.

For accounting purposes the primary ambition is to integrate environmental data with economic measures such as national income, gross domestic product and national wealth. This will address omissions in the System of National Accounts (SNA)^10^ because, the costs of environmental degradation, natural resource depletion, and non-market values of environmental services are not included. This is because the SNA focuses on goods and services transacted in markets, or perversely degradation is accounted for as income, as environmental losses often spur additional economic activity^11^.

In order to address this perverse outcome, where degradation is counted as an economic gain, we need to incorporate soil threats^12^ into an economic framework that recognises the cost to the economy of degradation caused by soil use and threats caused by human economic activity:

- *soil erosion by water*,
- *soil erosion by wind*,
- *decline in soil organic matter in peat*,
- *decline in soil organic matter in mineral soils*,
- soil compaction,
- sealing,
- contamination,
- salinization,
- desertification,
- flooding and landslides,
- decline in soil biodiversity.

If we are to treat soil as more than a non-depreciating growth substrate, and record both costs of soil degradation and the broader value its multiple life support functions provide, then we must measure the ‘state-and-change’ of soil resources. This can provide a basis for the implementation of policy instruments like payments for ecosystem services (PES) to restore the balance. The environmental accounts of the SEEA seek to link biophysical condition with economic activity to inform policy. The framework has the potential to address this measurement challenge and provide more complete measures taking into consideration, or adjusting for, environmental stocks and flows to inform policy. The lack of environmental information incorporated in GDP has led some to call for a complete end to the use of GDP^13^. This is unlikely in policy circles, and so extending the accounting framework appropriately to reflect environmental and economic information jointly seems a prudent approach. In this sense the European Commission has been a forerunner by presenting, in 2009, its road map towards moving beyond GDP (COM(2009) 433).

In order to achieve full resource accounting, environmental materials used in the economy must be identified. Often these are harvested or extracted, which is broadly recognised by the 7 asset classes used in SEEA-CF:

- Mineral & energy resources,
- Timber,
- Aquatic,
- Other biological,
- Water resources,
- Land cover,
- Soil.

As Obst^8^ points out, and as reflected in the research agenda for the SEEA, soils remain an under developed part of the SEEA-CF. Hence the aim of this paper is to bridge the science-policy interface by developing a natural capital accounting framework for soil that works for the SEEA-CF, but will also contribute to the developing SEEA-EEA^9^.

So how should we assess soil resources and how they change? There is a need to define the object (soil) and to choose an appropriate classification that will capture change in soil condition in response to use by an economic owner or climate change for example. In general soils are defined as, *any material within 2 m of the Earth’s surface that is in contact with the atmosphere*, *excluding living organisms*, *areas with continuous ice not covered by other material*, *and water bodies deeper than 2 m* ^14^. and are classified using either a natural systems approach such as the World Reference Base^14^ which divides according to morphology and genesis; or a technical classification system focusing on fitness for use, such as the Unified Soil Classification System used in engineering that recognizes mineral, organic and particle size as main criteria for division^15^. In addition, pedometric techniques are being developed that use statistical analysis to group soils, usually based on environmental properties or Jenny’s^16^ soil forming factors (Climate, organisms, relief, parent material)^Time^.

The current SEEA-CF proposes tracking soil materials such as carbon, nutrients and soil moisture in physical flow accounts, and classifying assets using one of the natural systems classifications. We propose that given most policy decisions are decided based on land use, rather than soil type, a more consistent approach for environmental-economic accounting is to use land cover for reporting rather than a soil classification. Countryside Survey in the UK^17^ uses land cover for reporting, but not collecting data, as this would confound statistical analysis of change^18^. Changes to soil stock, extent or condition are reported by land cover, and impacts of management interventions by land cover become more readily understood by policy. We propose a top level division by land cover, then subdivide soils according to their characteristics such as soil organic carbon, and potentially texture. Accounts can then be developed enabling the assessment of a soil property within a land use and how it changes with time.

We want to understand how the economic management of each land cover type impacts and changes the soil resource, rather than determining the suitability of a soil for a land use; which is often the function of soil survey. In contrast to SEEA-CF we therefore propose that soil extent, at this high level, is simply classified according to land cover, and that changes to volume or mass (e.g. erosion) and ultimately condition for ecosystem accounts are reported accordingly. SEEA identifies 14 land cover classes in its interim classification, but these could easily be expanded depending on the economic/policy activity of interest.

Biophysical asset accounts based on land cover, can then be populated using soil monitoring data which is seeing increasing investment on 5–10 year cycles to capture ‘soil condition change’ (UK Countryside Survey^17^; EU, LUCAS^19^; China^20^). Such an approach would begin to realize the proposal of Amundson^21^ who recently argued in Nature that assessment for maintaining soils in a good condition should be based on ‘*quantitative principles and measurements of soil erosion and production*, *soil nutrient loss and release*, *and soil carbon loss and return*’. While we agree with this, we also see the need for measuring how soil threats^12^ will impact the condition of the soil resource. We therefore propose an accounting framework and roadmap, using current EU data and identifying gaps to realise a functional set of environmental accounts for soils.

## Results

**Figure 1:**
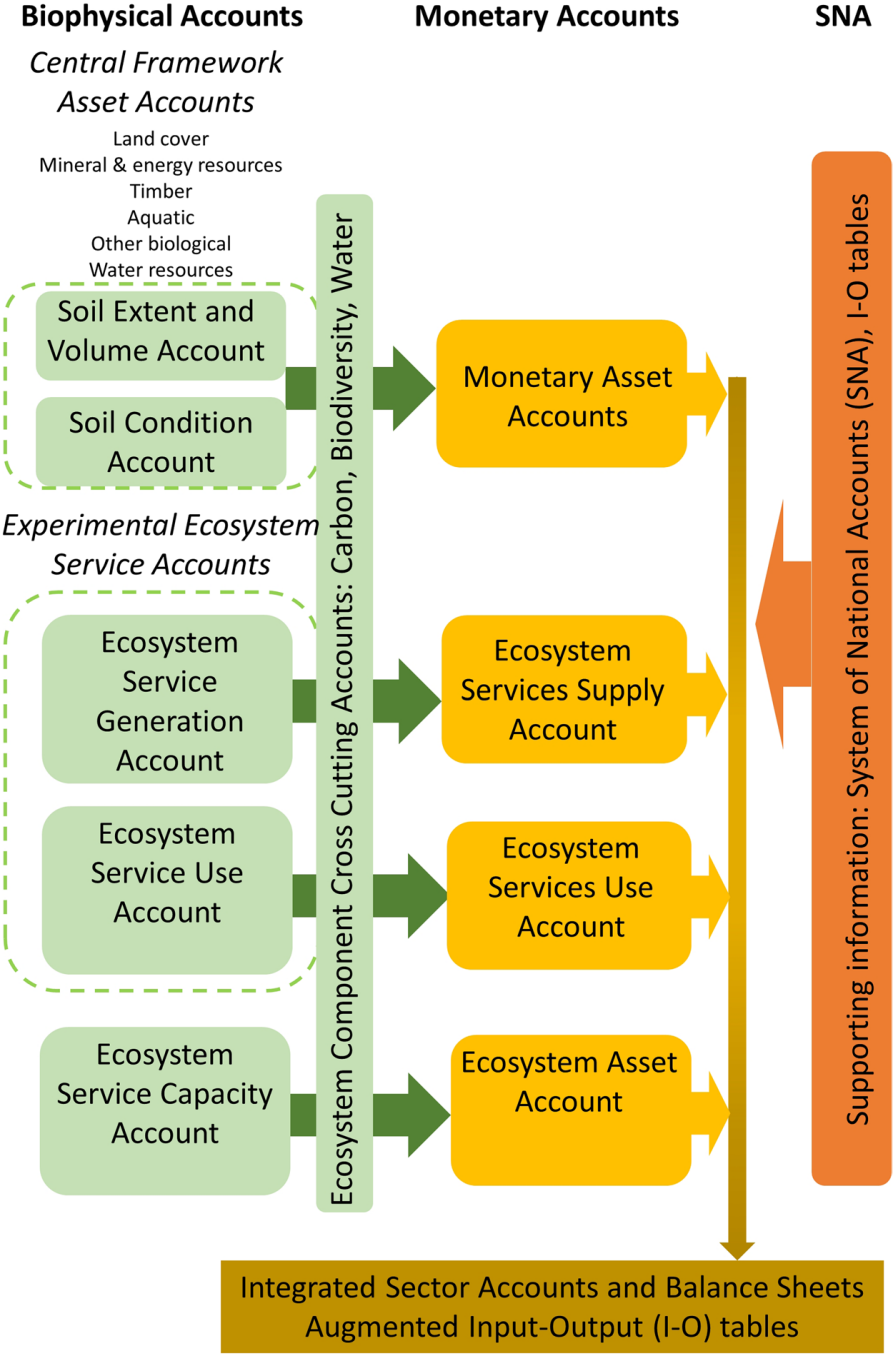
Conceptual overview of the SEEA accounting framework including the Central Framework^6^ (SEEA-CF) and the Experimental Ecosystem Service accounts (SEEA-EEA).

The general framework for SEEA accounting is presented in Fig. 1. The SEEA-CF proposes measuring the extent and condition of natural resources in seven asset categories, as bulleted previously. Ultimately the soil asset accounts will inform ecosystem service accounts, by providing a biophysical assessment of natural resources and a connection to economic and social activity. Economic contributions of the assets and materials can be converted to monetary accounts that can be used to augment input output (I-O) tables or form satellite accounts to the system of national accounts (SNA). Within the framework we propose soil extent is measured by land use. Sub division within a land use, e.g. grassland or woodland can be further applied according to soil properties such as soil organic carbon content or texture using property based mapping such as in LUCAS^19^ or SoilGrids^22^. This would ensure that soil change could be reported by land use, and in addition we will be able to report on the impact of land use change which accounts for a few percent of land.

The results presented here demonstrate:

- An example of land use classes based on an extent account using land cover data
- An example of a mass/volume account using soil production and erosion, and propose similar accounts for important soil cycles
- We discuss the need for accounts that include the impact of soil threats on soil condition

### Land cover change 2000–2012

**Figure 2:**
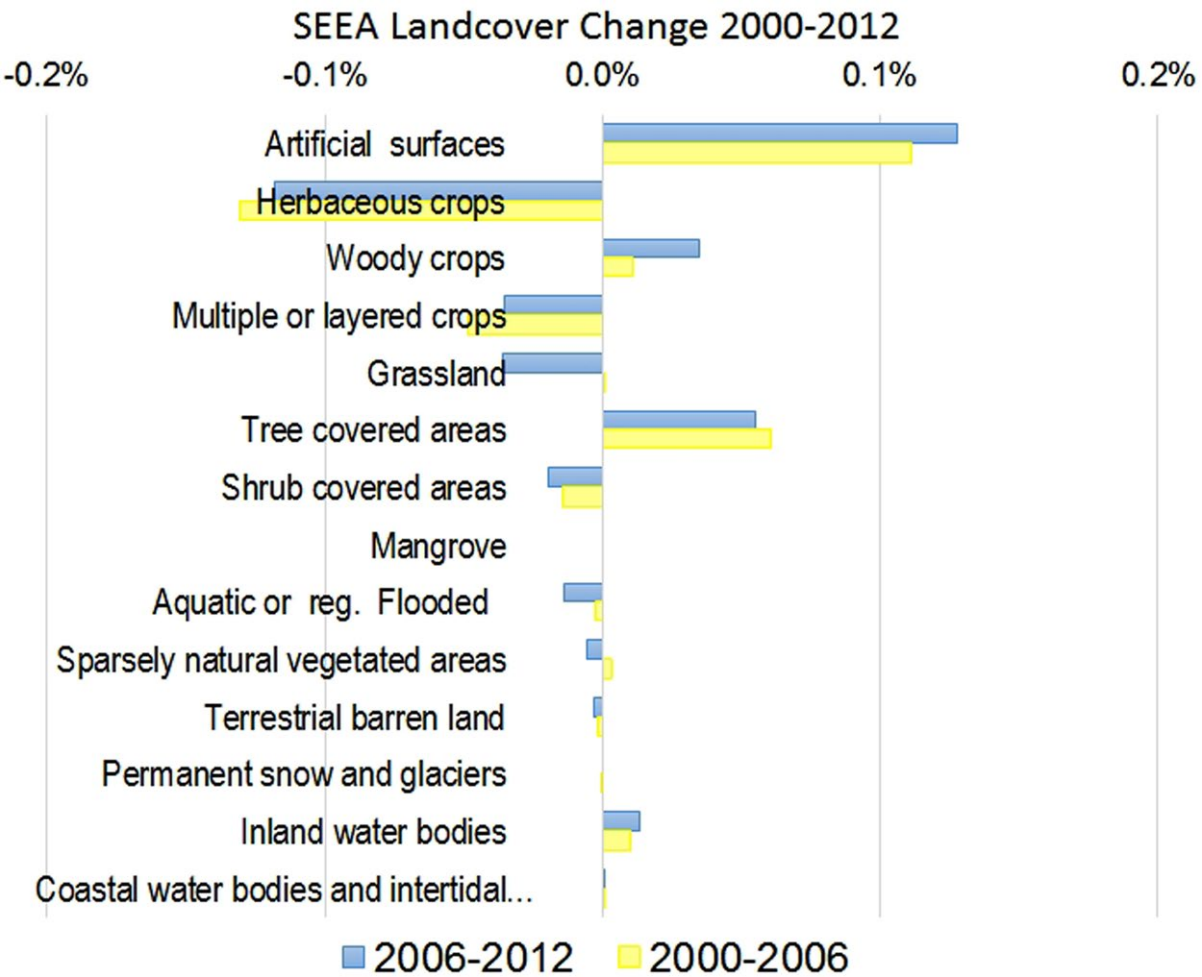
European land cover change according to the SEEA aggregate classes between 2000 and 2012. The % is relative to the total land cover in the EU-25 studied.

A new EU land cover map derived from the 44 Corine land cover classes^23^ aggregated into the 14 SEEA land cover classes can be found in the supplementary information (Fig S1 for map and Table S1 for the aggregation). Figure 2 is an assessment of land cover change in our LUCAS EU25 study area, derived from the aggregated Corine land cover change data. A major decline in herbaceous cropland occurred over the 12 year period, at a similar rate in each time period. Increases of similar size are observed in artificial areas, largely associated with urban sprawl. Tree cover has increased, both in terms of woodland and orchards. However, the aggregated figures also hide some substantial changes, for instance the change from coniferous forest (Corine-312) to transitional woodland scrub (Corine-324), which between 2000 and 2006 accounted for 35% of change across the EU28^23^.

Table 1 represents an attempt to produce an extent account table for EU land cover change between 2000 and 2012. It follows the proposed SEEA format. Additions to stock are divided into managed and unmanaged with artificial surfaces and cropped systems placed in the managed. Woodlands are hard to divide at this level, some are managed and some not, so only the change is recorded as with other semi-natural ecosystems.

**Table 1:** Extent account for change in land cover assets between 2000 and 2012 for the EU25.

SEEA Classes	1	2	3	4	5	6	7	8	9	10	11	12	13	14
	Artificial surfaces (incl. urban and assoc. areas)	Herbaceous crops	Woody crops	Multiple or layered crops	Grassland	Tree covered areas	Mangrove	Shrub covered areas	Shrubs and/or herb. veg., aquatic or reg. flooded	Sparsely natural vegetated areas	Terrestrial barren land	Permanent snow and glaciers	Inland water bodies	Coastal water bodies and intertidal areas
CORINE classes	1x	21x	22x	24x	231, 321	31x, 324		322, 323	41x	333	331, 332, 334	335	51x	42x, 52x
**Opening area km** ^**2**^ **of resources (2000)**	99,128	941,477	88,041	410,037	383,142	1,270,472	NA	143,195	76,508	17,298	5,054	1	55,996	2,106
**Additions to stock**														
Managed expansion	8354		1596											
Natural expansion														
Upward reappraisals														
*Total additions to stock*	8354		1596			4049						0	815	19
**Reductions in stock**														
Managed regression		−8676		−2921										
Natural regression														
Downward reappraisals														
*Total reductions in stock*		−8676		−2921	−1228			−1186	−565	−74	−182			
**Closing area km** ^**2**^ **(2012)**	107,482	932,801	89,636	407,116	381,913	1,274,521	NA	142,009	75,942	17,225	4,872	1	56,811	2,125

### Soil state and change

From the policy perspective we need to know the state of soils and how this changes due to degradation and policy intervention to reduce it. Monitoring soil cycles that impact the economy, society, and earth system function is crucial to this ambition. These include the carbon cycle, nutrient cycles, soil production and erosion cycle, and the water and energy balance. Moreover, soils are susceptible to change, and potentially degradation by drivers such as land use and land use change, climate change and pollution. One of the goals of the soil asset accounts is to assess the impact of these drivers of change on the condition of soil resources and ultimately the economy. This would realise one of the goals identified by the Inter-Governmental Technical Panel on soils^24^. The EU LUCAS survey is a ‘state-and-change’ monitoring survey that includes soils. Table 2 synthesises the available current, and proposed LUCAS data, indicating when future surveys will be conducted. We propose this is organised into four fundamental soil biophysical accounts that monitor these vital cycles:

**Table 2:** LUCAS soil measurements and their relation to primary soil cycles, threats and when they are to be sampled; modified from ref. 69.

Soil para-meters measured in LUCAS	Soil Cycles				Soil Threats											LUCAS sample Year				
	Carbon cycle	Nutrient cycle	Water and Energy balance	Soil for-mation/erosion	soil erosion by water,	soil erosion by wind,	decline in soil organic matter in peat,	decline in soil organic matter in mineral soils,	soil comp-action,	sealing,	conta-mination,	salini-zation	deserti-fication	floo-ding and land-slides	decline in soil biodi-versity	2009	2015	2018	2021	2024
Soil Organic Carbon (SOC)	+	**+**		+	#	#	#	#	#						#	++	++		++	
Soil Inorganic Carbon (SIC)	+	+														++	++		++	
pH	+	+					#	#			#	#				++	++		++	
Texture, and coarse fragments	+	+	+	+	#	#	#	#	#							++	++			
NPK		+														++	++		++	
CEC		+														++	++		++	
EC		+										#					++		++	
Sulphate sulphur, Na		+									#								++	
Heavy metals											#					++		++		++
Nitrate Nitrogen		+									#							++	++	
Organic Pollutants											#							++	++	
Thickness of peat	+				#	#													++	
Soil erosion	+	+		+	#	#													++	
Soil bulk density	+	+			#	#			#					#				++	++	
Soil moisture	+	+	+		#	#			#					#				++	++	
Soil biodiversity															#			++	++	
Land cover									#	#	#	#	#	#		CORINE DATABASE				
Extent accounts									*	*	*	*	*	*	*					
Mass accounts	*	*	*	*	*	*	*	*												

Extent and volume/mass accounts for soil cycles:

- Soil production and erosion
- Soil carbon gain and loss
- Soil nutrient release and loss
- Soil water and energy balance

Furthermore there is a need to capture the impact of soil threats on soil condition. Of the ones identified in the introduction the ones in italics are already covered by the soil accounts proposed above.

### Soil stock, production and erosion

**Figure 3:**
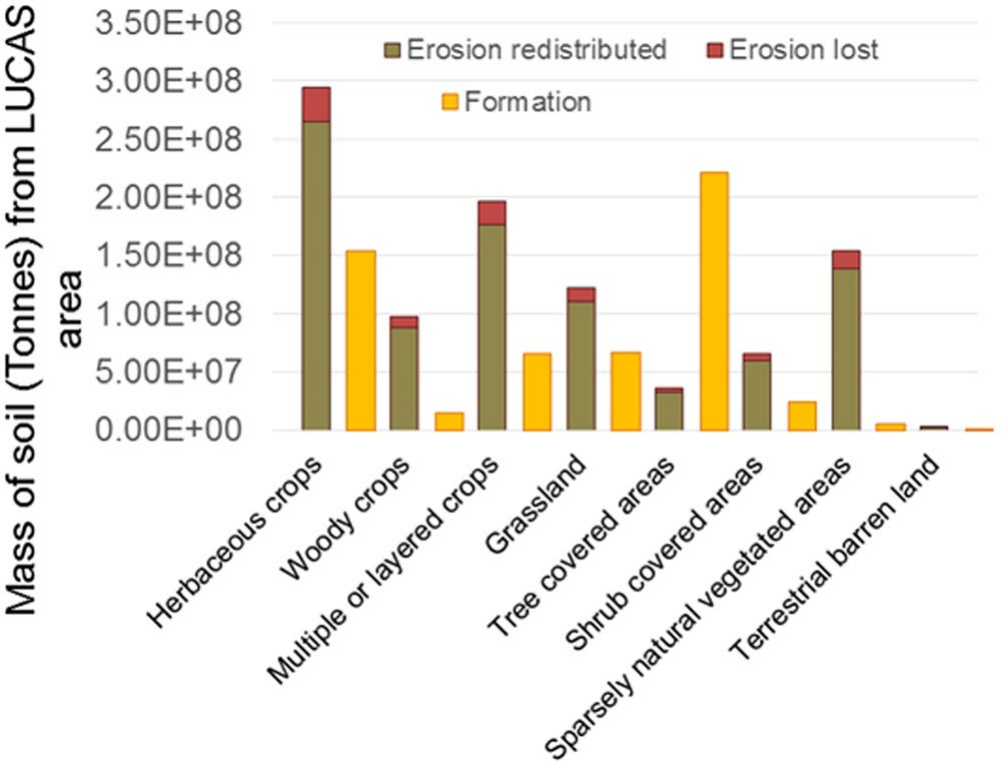
Estimated soil erosion across the EU-25 study area, with an attempt to divide the erosion into that which is redistributed on land and that which ends up in surface water and ultimately the ocean. Soil formation is estimated based on a potential upper bound for formation of 1.4 (t ha^−1^) based on Verheijen *et al*.^30^.

Land use change data is available for 2000 and 2012 which we use to produce an example soil stock account. Potential soil erosion rates are calculated for each SEEA land cover class based on spatial data analysis with the RUSLE2015 model^25^. Potential erosion rates are highest on land covers with sparse or no vegetation, but also on woody crop areas such as olive groves^26^. When multiplied up according to land cover area the mass of soil lost (tonnes ha^−1^) is estimated (Fig. 3). Erosion is the key parameter of interest so we can determine potential loss of carbon and nutrients from topsoil, estimating soil production is of interest if we want to estimate total soil loss. Very little data on soil production, especially at national and European scales is available. However, Riggins^27^ produced an average estimate of soil production of 15 m Million yr^−1^, with a residence time of ~30 000 yrs for soils generated from granite in the UK using ^10^Be. This equates to soil formation of ~0.15 mm yr^−1^ and soil production rate of 1.6 t ha^−1^. The soil production rate will vary with lithology, climate and soil depth^28^. These factors determine whether soil production rates follow either an exponential or humped relationship^29^. The work by Riggins^27^ demonstrates that the value of 1.4 tonnes ha^−1^ used as an estimate of the maximum expected level of soil formation in each land cover class^30^ within Fig. 3 is reasonable.

Land with sparse vegetation is highly erodible. In arable cropland areas erosion is consistently higher than production, a concerning trend^31^. For example, soils growing winter cereals and sugar beet have been identified as having the highest erosion rates in the UK^32^. However, the loss of soil is potentially higher in some cropping systems where bare soil is present for periods during crop establishment and after harvest, coinciding with greater seasonal precipitation and lower evapotranspiration. Examples are those of maize where acreage is increasing due to use as fodder and bioenergy production^33^ and willow, which is used as a bioenergy crop but leaves soil bare during the establishment phase^34^. However, in comparison to winter cereals these non-arable crops occupy relatively small areas, but may have extended their climatic range, potentially now growing in areas of higher annual precipitation. The establishment of grass can also lead to high erosion rates during the early stages, as a result of it sometimes occupying steep slopes, and in areas of higher precipitation. Techniques to measure soil erosion all have potential weaknesses including edge effects on erosion plots^35^ or those associated with ^137^Cs and therefore must be considered as estimates of magnitude. Although erosion is high in croplands (for example typical estimates for the UK using ^137^Cs as an erosion tracer are between 0.01 and 10 t ha^−1^ a^−1^; with a median of ~2 t ha a^−1^, across all different slope steepness and lengths tested^36–41^), recent research suggests that interventions (e.g. use of cover crops, increasing SOM and aggregate stability, adopting conservation agriculture practices of no-till or reduced tillage), are having an impact reducing erosion^42^. It is important to note that erosion is hard to assess^35^ and methods such as ^137^Cs have been criticised^43–45^, so it is important to acknowledge potential uncertainty in the magnitude of the numbers. However, it is reasonable to point out that tree covered areas are distinctive with soil production estimated to outpace erosion by as much as five times, therefore, maintaining native woodlands protects soil.

Table 3 shows how this data can be presented in an accounting format with changes between 2000 and 2012. The upper part contains the additions to the stock, while the lower part the reductions. The numbers in the table are uncertain and although providing a framework and potential way to account, highlight the uncertainty regarding additions and reductions. We don’t have spatially representative numbers for soil production, so we have used upper and lower bound estimates (0.4–1.4 t/ha) based on Verheijn *et al*.^30^. Soil erosion estimates are based on the RUSLE2015 approach^25^ and limited to rill and sheet erosion determined by land cover class. Of the soil that is eroded a poor understanding exists of how much is lost to water courses and how much is redistributed over the land. The difference between the sediment deposited and the throughput of eroded material is referred to as the ‘Sediment Delivery Ratio’ (SDR). For example, the quantity of soil being delivered to the fluvial system has been defined as the ratio of sediment delivered at the catchment outlet (t km^−2^ yr^−1^) compared to gross erosion rates (t km^−2^ yr^−1^) within the basin or catchment^46^. However a major control on sediment delivery is catchment size. Porto, *et al*.^47^ explored this concept on three catchments of different sizes in southern Italy. The results of this study demonstrate a reduction in the sediment delivery ratio from 98 to 2% as catchment area increases from 1.47 ha to 312 km^2^. Knowing sediment delivery is important for planning activities such as dredging for navigation and flood prevention.

**Table 3:** Mass account for soil stock, prototype biophysical account table for EU soil formation and erosion between 2000 and 2012.

SEEA Classes	1	2	3	4	5	6	7	8	9	10	11	12	13	14
	Artificial surfaces (incl. urban and assoc. areas)	Herbaceous crops	Woody crops	Multiple or layered crops	Grassland	Tree covered areas	Mangrove	Shrub covered areas	Shrubs and/or herb. veg., aquatic or reg. flooded	Sparsely natural vegetated areas	Terrestrial barren land	Permanent snow and glaciers	Inland water bodies	Coastal water bodies and intertidal areas
CORINE classes	1x	21x	22x	24x	231, 321	31x, 324		322, 323	41x	333	331, 332, 334	335	51x	42x, 52x
**Opening stock of resources (2000)**														
**Additions to stock**														
Soil formation (1.4 tonnes/ha)		154,650,859	14,417,942	65,302,806	66,705,312	221,286,551		23,817,473		5,300,489	164,366			
Soil formation (0.4 tonnes/ha)		44,185,960	4,119,412	18,657,945	19,058,660	63,224,729		6,804,992		1,514,426	46,962			
Soil deposition (From erosion)		265,447,183	87,767,154	176,760,582	110,417,456	32,304,933		59,206,778		138,881,709	2,208,581			
Upward reappraisals														
Reclassifications														
*Total additions to stock*		420,098,043	102,185,096	242,063,388	177,122,768	253,591,484		83,024,250		144,182,199	2,372,946			
**Reductions in stock**														
Extraction (construction) sealed?	?													
Soil eroded redeposited		265,447,183	87,767,154	176,760,582	110,417,456	32,304,933		59,206,778		138,881,709	2,208,581			
Soil eroded lost to water courses		29,494,131	9,751,906	19,640,065	12,268,606	3,589,437		6,578,531		15,431,301	245,398			
Downward reappraisals														
Reclassifications														
*Total reductions in stock*		294,941,315	97,519,059	196,400,647	122,686,063	35,894,370		65,785,309		154,313,010	2,453,978			
**Closing stock (2012) 1.4**		125,156,728	4,666,036	45,662,741	54,436,705	217,697,114		17,238,942		−10,130,812	−81,032			
**Closing stock (2012) 0.4**		14,691,828	−5,632,494	−982,120	6,790,054	59,635,292		226,461		−13,916,875	−198,436			

Note that soil erosion estimates are limited to rill and sheet erosion and are likely a lower bound.

The assessment of closing stock indicates how important it is to obtain more accurate numbers for this mass balance; (Table 3) soil production, erosion, redistribution and loss. It appears clear that sparsely vegetated areas and barren land are subject to soil depletion. While it is unclear if cropped areas are permanently gaining or losing soil, determining the rates at which this is happening is difficult. Further information is also required as to how different land use practices impact this balance at the EU scale.

### Soil carbon gain and loss

**Figure 4:**
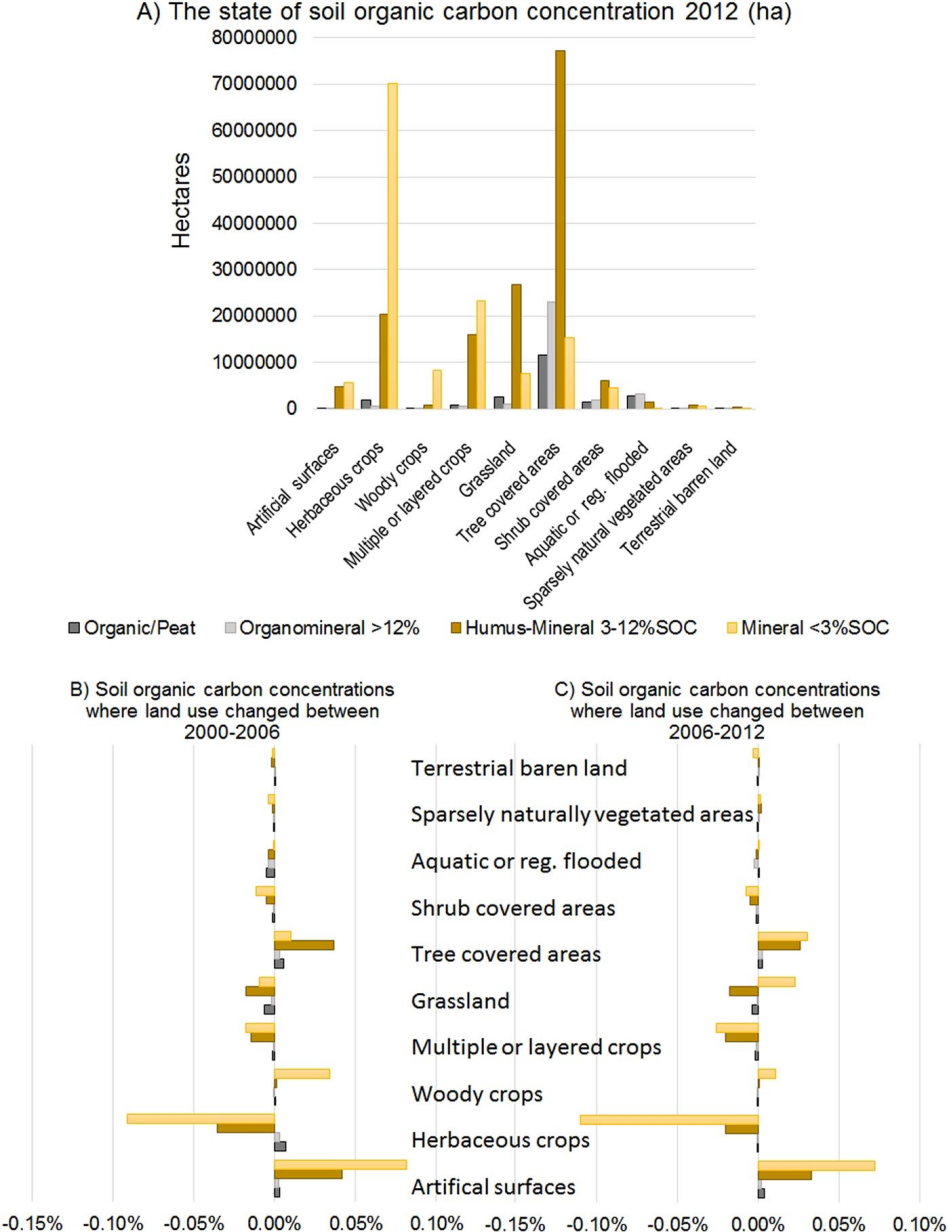
(**A**) The area of land with respective soil organic carbon concentration, by land use (ha) in the EU-25 study (carbon concentrations divided into 4 categories using the LUCAS data). (**B**) The soil carbon concentration of land subject to change of use between 2000 and 2006. (**C**) The soil carbon concentration of land subject to change of use between 2006 and 2012.

Soil organic carbon (SOC), which includes peat, is increasingly seen as an important measure of soil condition, supporting biogeochemical and physical function; LUCAS 2009 contains data on the state of carbon concentration (g/kg), change data will not be available until the 2015 data is analysed in 2017 and data become available in 2018. So we can’t know how carbon levels are changing within land uses yet, but we can assess the state of soil resources by land use according to carbon concentration (organic, organo-mineral, humus-mineral and mineral). Moreover, knowing the land cover change, we can at least see which soils are most vulnerable to habitat change, understanding management impacts will only come later as subsequent repeat data is analysed. Figure 4A shows extent (ha) of each SOC group based on the 2012 land cover class. Herbaceous crops and tree cover are the dominant land covers with trees being located on soil resources with higher SOC carbon content. Trees are also the dominant cover on organo-mineral soils. By comparison wetlands with organo-mineral soils >12% SOC excluding peat, occupy an area of ~3.3 million ha, while woodlands cover ~23 million ha, ~7 times greater, indicating the need to conserve these woodland areas deforestation means a potential double hit of loss of both wood and soil carbon.

Cropping activity, whether herbaceous, woody or multiple layered crops predominantly occurs on mineral soils. Evidence from both the Countryside Survey in the UK^17^, and National Soil Inventory^48^ indicates a decline in arable topsoil carbon concentration between 1978 and the last CS survey in 2007. This is in agreement with EU country level studies reported in Reynolds *et al*.^17^ (Table 7^17^). Conversely, woody crops, e.g. olive plantations are often planted on the steep low fertility soils and are likely naturally low in SOC.

Development of carbon accounts is hampered by not yet having the soil temporal change data. We can however see which soils are being impacted by land cover change, although this is only a small component of the potential overall change. By combining the land cover change data and soils data we can assess which soil resource groups are most subject to land cover change at EU scale (Fig. 4B and C). Between 2000 and 2012 about 2/3 of the increase in artificial surface occurred on low SOC mineral soils, but 1/3 occurred on humus-mineral soils. The decline in herbaceous crops was predominantly on mineral soils as was the increase in woody crops. Increases in woodland occurred predominantly on humus mineral soil in the first 6 years, switching to mineral soils in the latter 6 years. Decreases in organic soils appear to be more pronounced in the aquatic, grassland and shrubland covers up to 2006, appearing to slow after this time up to 2012. There was an increase in organic soil being cropped between 2000 and 2006 which appears to have halted after 2006.

The data forms the basis of developing biophysical accounts like Table 1, with 70% of points being resurveyed in subsequent monitoring to assess change. For full carbon accounting both SOC and soil inorganic carbon (SIC) are required, which LUCAS contains. Determination of the carbon stocks will not be feasible until the 2018 data is collected with bulk density (Table 2). Even then interpretation may be challenging without a complete picture of where peats are located. Future surveys (2021, Table 2) will rod for peat depth to improve assessment but there is no doubt that improved extent and volume mapping of peat is required, which given developments in airborne assessment methods^49^, is becoming feasible.

### Soil nutrient release and loss

**Figure 5:**
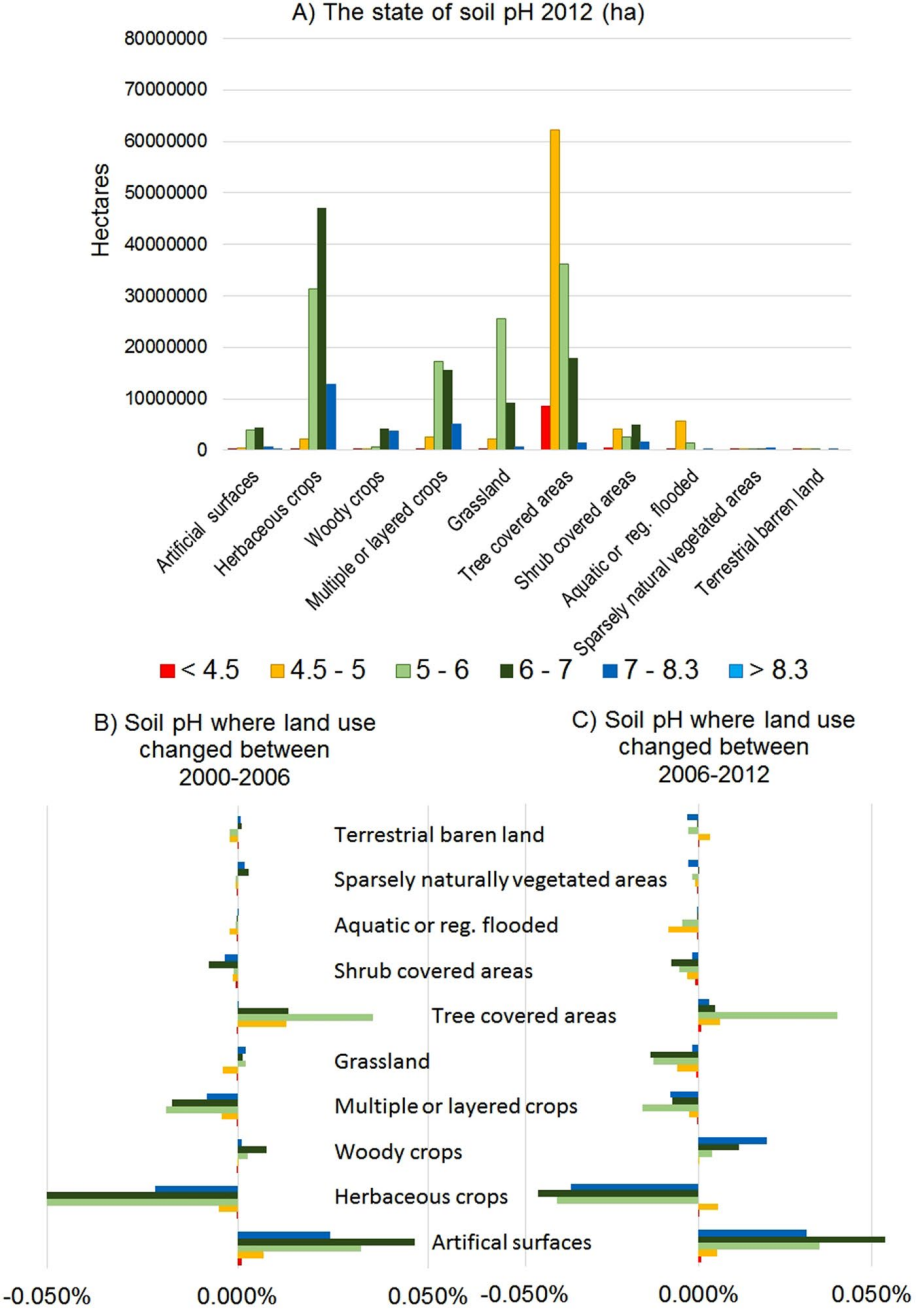
(**A**) The area of land (ha) in the EU-25 study area with pH divided into 6 categories using the LUCAS data. (**B**) The pH of land subject to change of use between 2000 and 2006. (**C**) The pH of land subject to change of use between 2006 and 2012.

LUCAS contains data on total nitrogen, potassium and available phosphorus^19^. Here we focus on soil pH as a general indicator and integrator of the nutrient release environment and because pH is an indicator of some soil contamination (acid deposition) or salinization (pH > 8.3). The state of soil pH is presented in Fig. 5A, and shows the major difference between the cropped areas including grassland, and woodland and areas of semi-natural vegetation. The data indicate a gradient with herbaceous crops dominating on the neutral and slightly alkaline soils (6–7), while the grasslands are on the neutral soils (5–6) and the woodland extensively covers low pH, acidic soils (4.5–5). We see that the artificial surfaces cover neutral soils which means these soils are lost to production. Figure 5B and C allow us to see where the changes are occurring. Major changes are occurring on crop lands with consistent declines in neutral and slightly alkaline soils. Artificial surface and tree cover is expanding, the artificial surface taking much of the arable land, while tree cover is expanding on neutral soils.

Nutrient cycling is vital to developing sustainable growing systems^50^. In addition to the nutrient retention data in LUCAS, full accounting will need data on organic amendment and manufactured fertiliser additions and management. If estimates of crop uptake and erosion loss can be estimated then leaching losses might also be determined, helping to tighten up nutrient cycling.

### Soil water and energy balance

Soil moisture and the energy balance contribute to biomass production, climate, hydrological and disease regulation. Our ability to measure moisture and temperate is increasing with Satellite observations e.g. sentinel I and II, ground based COSMOS and point sensors^51^. We need to determine how to assess it over time, perhaps measuring the soil moisture distribution, to better link to models^52^. Ultimately, the ability to account for changes in the soil moisture probability density function will allow us to better assess the social and economic impact of extreme weather such as drought, floods and heatwaves.

### Extent accounts that include the impact of soil threats on soil condition

Soil erosion by water and wind and the decline of organic matter in peat and mineral soils can be assessed through the proposed soil production and erosion, and carbon gain and loss accounts. With regards to the other soil threats the development of state and change extent accounts, like the land cover account could form the basis of initial accounts. Knowing the state and change in terms of areas affected by soil threats is an important contribution to informing policy. The extent of a number of the soil threats could be determined from remote sensing data e.g., sealing, desertification, flooded area and landslide occurrence. Others like compaction might be informed by modelling like we’ve done for erosion, informed by data measured in the LUCAS survey. While the extent of contamination, salination and biodiversity will need to be assessed from LUCAS measurements. At present DNA analysis would simply provide an assessment of biodiversity, essentially natural history. However this is still an emerging field that has been revolutionised by microbial genomic methods^53^, As we progress from meta-barcoding methods used to assess biodiversity, to other omic methods used to assess the quantity of functional genes, proteins or metabolites we will see a revolution in our understanding of soil processes. Proof of concept accounts using such omic approaches, as well as traditional count methods are now available, as number of species per km^2^, and relate to the resilience function (appendix A^54^). What we stand to learn is exemplified in ref. 55 and 56. Not currently included in LUCAS, the intention is to bring it in in the future (Table 2).

## Discussion

The SEEA framework encompasses two approaches to accounting for soil resources. The first, described in the SEEA-CF^6^, treats soil as an “individual” resource alongside other environmental assets. In effect the logic presented is to consider that soil is a resource that can be measured and accounted for in an isolated, standalone fashion.

The second approach is described in the SEEA Experimental Ecosystem Accounting (SEEA-EEA)^9^. This approach does not seek to account for resources as separable assets but rather aims to consider how environmental assets combine within spatial areas – ecosystem assets - to deliver ecosystem services of benefit to economies and society generally. This approach builds on the significant advances in the measurement and assessment of ecosystem services such as in the landmark Millennium Ecosystem Assessment^57^, the TEEB^58^, CICES^59^ and, in the UK, the National Ecosystem Assessment^60^.

This discussion describes how both of these frameworks can be applied to provide a platform for integrating a range of soil resource information. Before providing this description it is relevant to understand how soil resources have traditionally been considered in national accounting frameworks. The standard approach to measuring the economy, including measures such as GDP, is described in the System of National Accounts (SNA)^10^. The SEEA adapts the standard accounting principles and structures of the SNA to support the integration of environmental information with standard economic data.

Concerning soil resources, the SNA is effectively silent. Although it does cover accounting for various natural resources, soil is inseparable from land – in effect the value and role of soil is not distinguished from the location and context factors that drive land values. While this may be sufficient for the purposes of estimating the monetary value of, for example, agricultural land, for inclusion in measures of national wealth, it is distinctly insufficient for the purposes of understanding the physical impact of physical changes in the condition of soil resources and for bringing the broad public benefits from soil (e.g. carbon storage) into the picture.

To this end, in the development of the SEEA-CF, while there was little experience in the measurement of soil resources among the national accounting and statistical communities developing it, soil was distinguished from land as a distinct resource and the development of accounting for soil resources was placed on the SEEA research agenda.

In the development of the SEEA-EEA, there was some discussion on soil but largely it was characterised as being an inseparable part of an ecosystem asset which was, in broad terms, delineated by virtue of different land covers (forests, wetlands, grassland, crops, etc). While soil condition was seen as a potentially important aspect of understanding the condition of an ecosystem asset, most ecosystem services from soil were not considered of direct benefit to economic units and society, but rather were seen as supporting or intermediate services. The value and role of soil as soil, thus became hidden^61^.

The SEEA-CF Framework focus on soil resources as individual assets is useful for recording physical changes in the stock of soil resources such as through soil erosion, deposition, or soil production. In concept, an account for soil resources would record a volume or mass of soil resources in cubic metres or kg at the beginning and end of a reporting period and then account for changes in the volume or mass, and ultimately its condition. In practice, understanding the condition of the resource is central, and something not addressed in the SEEA-CF, reflecting the characteristics of soil. Condition metrics are most useful for analysis and policy; hence our proposal for biophysical account tables based on cycles and condition. The largest drawback with even this simple framing of soil resources is that the SEEA-CF does not highlight the relevance of where soil is located in the landscape. Providing a national level volume of soil resources may sound appropriate but is impractical and not particularly helpful, and would be hard to assess. The reality is that the location of soil resources, and how soil resources in those locations change over time, is really the important issue. The framework proposed addresses this, and provides an assessment of the soil asset based on mass balance of the stocks that support vital functions, and the impact of any threats on the condition of the soil. This framework would therefore inform both the SEEA-CF and SEEA-EEA.

The underlying approach of the SEEA-EEA is to account for different spatial areas within a country. Although discussion continues on exactly how those spatial areas might be best delineated, the general approach is to, in a first step, divide a country into areas by distinguishing different vegetation types. A first proxy for this at an aggregated level is to use land cover as proposed here.

Initial discussions on ecosystem accounting highlighted the potential to also use soil type as a means of distinguishing different areas within a country but this approach did not gather support within the London Group, for some of the reasons stated earlier. Discussion continues with the proposal that soil and ecosystem assets be distinguished within the accounting framework to recognise the quite different ecological roles that soil and vegetation play. In practice, this would mean that ecosystems and soil resources would be accounted for separately by delineating spatial areas relevant to each resource type. We have proposed an alternative path with a top level division by land cover, then subdivision according to soil characteristics such as soil organic carbon and texture for example. The development of soil property maps such as those from LUCAS or globally from SoilGrids^22^, make this feasible. Thus we may begin to better understand how the mix of vegetation and soils results in quite different mixes of ecosystem services and the need to consider different management responses.

This adaptation of the current SEEA-CF framework would support the development of both maps of soil properties and maps of vegetation/ecosystem types. For soil resources specifically, recognising different soil property units within the landscape provides a basis for recording changes in the mass of soil resources, for example due to erosion, and soil condition under different management, between different locations within a country. This is a material improvement on the basic accounting table presented in the SEEA-CF.

One of the key aspects of ecosystem accounting described in the SEEA-EEA is the measurement of ecosystem condition. Ecosystem condition measures are intended to reflect the overall integrity and functioning of the ecosystem. They may also be considered measures of ecosystem health. The proposed approach to measurement in the SEEA-EEA is to collate information for a number of relevant ecological indicators considered to best reflect the ecosystem condition. It is expected that the set of indicators would vary by ecosystem type.

The extension to soil, building on the delineation of distinct spatial assets for soil resources as mentioned above, is to collate information about key characteristics of soil resources, e.g. carbon, texture, and pH that contribute to function, and condition indicators that would assess any impact of threats to that function. We argue that the proposed biophysical account tables for the cycles, 1–4, could form the basis of a minimum data set, which must be augmented by condition metrics. Measurement of these characteristics over time, in conjunction with land cover, across a country would provide a rich source of information on changes in the quality and health of soil resources, as impacted by management and environmental change, demonstrated by the periodic Countryside Survey in the UK^17^. In this context, accounting provides a platform for the organisation and presentation of relevant information for the socio-economic context.

With LUCAS and other EU monitoring programmes, plus the extensive experience in developing agri-environmental indicators^62^, Europe is well placed to develop pan European accounts extending beyond flows to include resources such as soil. LUCAS is an example of the type of modern soil monitoring system to detect state and change called for by the Intergovernmental Technical Panel on Soils^24^. Such a system has the potential to inform natural capital accounting frameworks to provide a fully integrated environmental-economic and social accounting system.

## Methods

### Land cover data

Spatial and temporal information on pan-European scale land cover change for the period from 2000 to 2012 were acquired from the CORINE Land Cover (CLC) inventory^23^. Raster data at 100 meters resolution were selected. Changes in land use were mapped using the complementary land change layers ‘LCC 2000–2006’ and LCC ‘2006–2012’, reporting changes in land cover with a minimum mapping unit of 5 hectares. The 44 land cover classes reported in the CLC maps were aggregated into the 14 SEEA scheme as illustrated in Table S1. Geospatial data processing and statistical analysis were carried out in ArcGIS 10.2 environment.

### LUCAS soil data and group criteria

Soils are divided into peats, determined from the EU peat map^63^. The peat is based on data which is synthesized from the European soil database v 1.0^64, 65^, to mask those 1 km areas with a probability of finding peat >35%. To date this is the best available EU peat map product. Everything that is not peat but has an organic carbon concentration >12% is classed as organo-mineral based on the LUCAS data^66, 67^ similar to IPCC 2006^68^ (Annex 3A.5, Chapter 3 in Volume 4). Mineral soils are subdivided into humus-mineral (3–12% SOC) and mineral (<3% SOC).

Soil pH was divided into 6 categories (<4.5, 4.5–5, 5–6, 6–7, 7–8.3 and >8.3). The divisions are mostly arbitrary to visualise the data. But the end values have physical significance. Soil with a pH <4.5 would be expected to have toxic levels of aluminium coming into solution, while in soils with a pH >8.3, boron toxicity can be a common feature along with dominance of sodium on the exchange complex resulting in soil structural problems.

## Electronic supplementary material

Supplementary information
