# Supplementary material for: Soil natural capital in europe; a framework for state and change assessment
Source: Sci Rep. 2017 Jul 27;7:6706. doi: 10.1038/s41598-017-06819-3 (PMC5532245; doi:10.1038/s41598-017-06819-3)
Supplement: Supplementary file 1 — Supplementary information [file 41598_2017_6819_MOESM1_ESM.pdf]

Supplementary information

Soil Natural Capital in Europe; A Framework for State and Change Assessment.

Robinson, David A.,[1] Panos Panagos[2], Pasquale Borrelli[2], Arwyn Jones[2], Luca Montanarella[2], Andrew Tye[3], Carl G. Obst[4].

1 NERC–Centre for Ecology and Hydrology, Deiniol Rd, Bangor, LL57 2UW United Kingdom
2 European Commission, Joint Research Centre, Via E. Fermi 2749, I-21027, Ispra (VA), Italy
3 NERC–British Geological Survey, Environmental Science Centre, Keyworth, Nottingham, NG12 5GG United Kingdom
4 Institute for the Development of Environmental-Economic Accounting (IDEEA) & Melbourne Sustainable Society Institute, University of Melbourne, Parkville, Vic, Australia

Table S1, aggregation of Corine classes into 14 SEEA land covers.

	SEEA class	Corine class
1	Artificial surfaces (incl. urban and assoc. areas)	1x
2	Herbaceous crops	21x
3	Woody crops	22x
4	Multiple or layered crops	24x
5	Grassland	231, 321
6	Tree covered areas	31x, 324
7	Mangrove	NA
8	Shrub covered areas	322, 323
9	Shrubs and/or herb. veg., aquatic or reg. flooded	41x
10	Sparsely natural vegetated areas	333
11	Terrestrial barren land	331, 332, 334
12	Permanent snow and glaciers	335
13	Inland water bodies	51x
14	Coastal water bodies and intertidal areas	42x, 52x

Figure S1, SEEA land cover map for the EU-25 corresponding to the area covered by the first LUCAS survey. The legend corresponds to the habitats in Table S1. The map is derived from the Corine data[1] overlaid on the Esri world terrain base map (relief/ocean map)[2] using ArcGIS 10.2 environment.

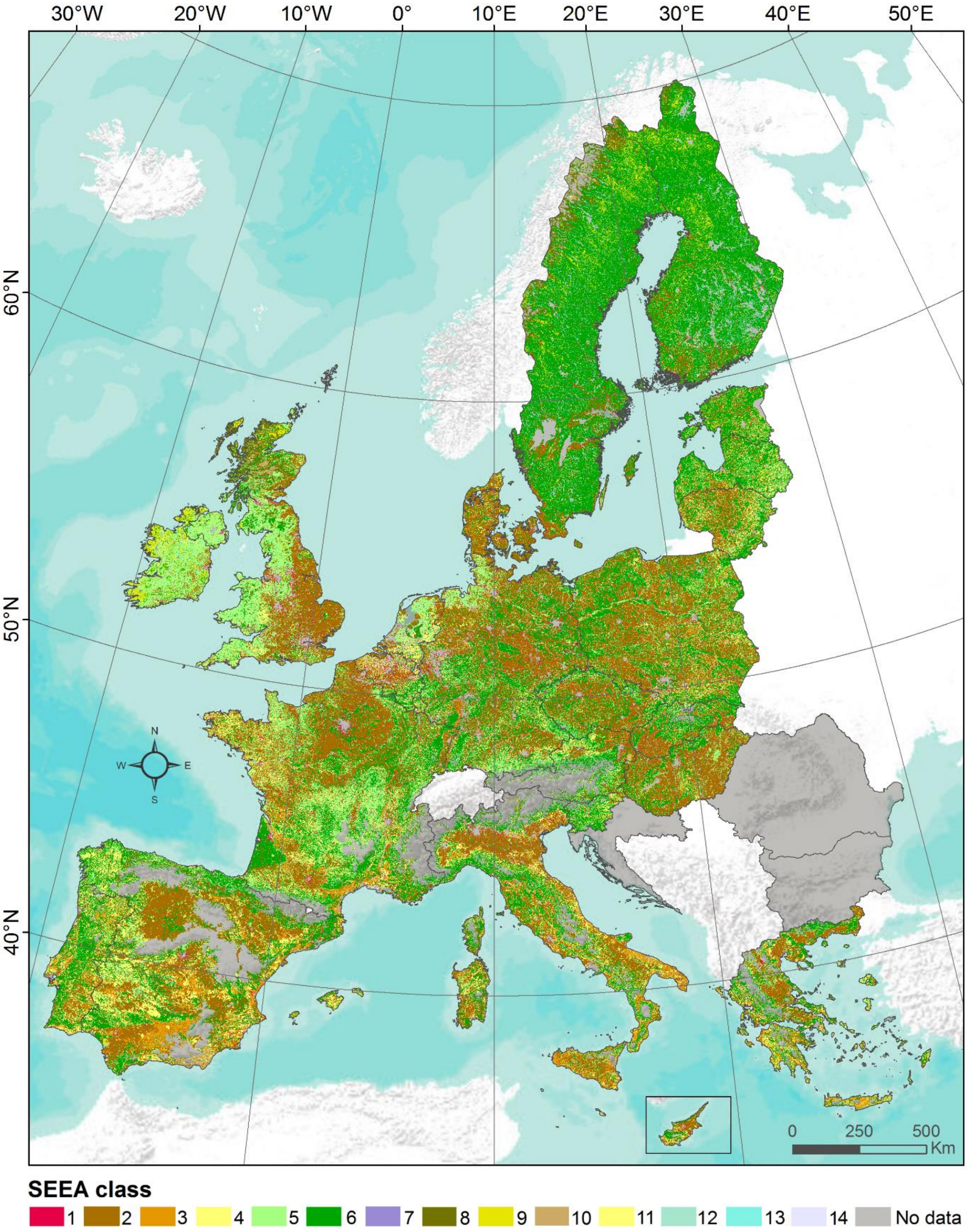

Measurement considerations for developing monetary estimates.

Ecosystem services

The final piece of the accounting framing in relation to soil resources is to make the connection to the rapidly growing measurement discipline of ecosystem services. In the SEEA-EEA core model, there are three key measurement components. The first is the delineation of ecosystem assets and the measurement of that ecosystem's condition. This supports understanding of how the ecosystem asset is changing over time and provides a basis for the measurement of the accounting concept of degradation as a cost of capital due to human activity.

The second is measurement of flows of ecosystem services, commonly differentiated between provisioning, regulating and cultural services. These services are considered to flow to economic units, households and society generally. In some cases they are inputs to goods and services that are already recorded in standard economic accounts (e.g. crops, timber). In other cases they reflect additional consumption, for example in terms of flood mitigation provided by local forests. The analogy to standard accounting is that each ecosystem asset is effectively a new producing unit (like a factory) that supplies ecosystem services as outputs through the application of ecosystem processes. Its capacity to supply these services into the future will be directly related to the condition of the ecosystem.

The third component is accounting for the benefits obtained. In the SEEA-EEA, ecosystem services are defined as "contributions to benefits" thus making a distinction between flows of ecosystem services (e.g. air filtration) and the resulting outcomes (e.g. clean air). This can be quite significant in terms of valuation since different considerations will be relevant in assessing the value of clean air as distinct from the role of ecosystems in contributing to this outcome.

As noted above, the SEEA-EEA framework does not currently extend to considering soil as a distinct asset but, at least conceptually, the ecosystem accounting model just described could be easily adapted to reflect such a change. This change would see both an ecosystem asset (i.e. the above ground flora and fauna combination) and a soil asset being accounted for as distinct producing units. The soil asset would then be considered to supply 'soil services'.

The additional extension to the current SEEA-EEA model that is required is to incorporate flows of intermediate or supporting services. These would arise when a soil asset provides services to an ecosystem asset rather than direct to an economic unit or household. In the overall accounting system, the recognition of intermediate service flows between environmental assets does not affect the final net result in terms of overall impact on the

economy and society – i.e. what is an output for a soil asset is an input of equivalent magnitude for the ecosystem asset. However, by separately recording soil services, the role of the soil asset can be identified within the broader ecological processes, which is often critical information for policy development. This in turn provides the basis for a more complete assessment of soil resources, since a connection can then be made between the observed physical condition of the soil and the flow of soil services it supplies and the flow on connections through the extended supply chain similar to the concept proposed in Dominati et al.[3].

Through the description of the SEEA's accounting framework, the focus is largely on the relationships between different soil related variables, both stocks and flows, and the different ways in which these might be integrated. This framing thus makes no initial statement as to whether estimation should be conducted in physical or monetary terms. Put differently, SEEA based accounting does not require data to be converted into monetary terms. A coherent way of organizing information across environmental asset classes to understand state and change is in itself a valuable contribution to policy development and decision making.

Valuation

The key message from considering valuation for accounting purposes is to recognise that not all estimates expressed in monetary terms can be used in all situations. Different valuation techniques and assumptions will all derive a number in a given currency (dollars, pounds, euros, etc) but may reflect quite different valuation concepts. Comparing estimates based on different valuation concepts is inappropriate and particular care should therefore be taken in (i) determining the focus or purpose of valuation; (ii) selecting the relevant valuation concept; and (iii) choosing appropriate valuation techniques.

For accounting purposes the primary ambition is to estimate values that can be integrated with existing measures of economic activity, such as GDP and national wealth, and hence provide more complete measures that take in account or are adjusted for environmental stocks and flows, for example degradation adjusted measures of GDP. With this in mind, the valuation concept applied in the SEEA is "transaction prices", i.e. based on prices for environmental assets and services that would apply if the associated asset or service was exchanged between two units, where for example ecosystem assets can also be considered a unit.

An alternative valuation purpose is to assess the overall, societal value of environmental assets. Another valuation purpose is to compare potential differences between the welfare outcomes that arise under different policy choices (e.g. different tax regimes). Each of these different valuation purposes will require the use of different valuation concepts and different

valuation techniques. In general, the information on physical stocks and flows recorded in an accounting setting can be used to underpin all valuation purposes and to this extent the SEEA provides a multi-purpose framework to support different valuation work.

There is a range of valuation techniques that can be used to estimate transaction prices. Of most relevance in relation to soil resources are production function methods, hedonic pricing, resource rents and replacement cost approaches. In all cases, these approaches use information on transactions in marketed goods and services to derive the price of an associated service. For example, the prices of soil services involved in the production of wheat could be estimated on the basis of wheat prices.

Following the logic of ecosystem accounting, the value of a soil asset is estimated by valuing each of the different soil services provided by the asset, projecting the future flows of these services (dependent on the forecast condition of the asset), and obtaining the net present value of the future income stream. This approach ensures that all of the relevant ecosystem services, including those of general public benefits, are included in the asset value.

An alternative, for some soil assets, may be to utilise information on sales of land, particularly agricultural land. The sale prices should, in theory, provide an indicator of the value of the associated soil. However, (i) it will be difficult to determine the value of soil that is inherent in the price of agricultural land from other factors; and (ii) observed land prices are unlikely to provide a valuation that encompasses the public benefits of soil services. Nonetheless, agricultural land prices may provide a useful starting point and check on the estimation of soil asset values for specific soil services.

Research is ongoing to determine appropriate valuation techniques for different soil services for use in an accounting context. Work to date, e.g. World Bank[4], reveals that many methods currently used for other purposes are likely to be appropriate when the services supplied are inputs to existing economic activity or can be adapted to provide transaction prices.

Irrespective of the valuation concept applied, the largest challenge in valuation is estimating prices and values that taken into account local circumstance given that the interactions with the environment do not involve actual exchanges of money. While no money is exchanged, it is important to recall that, at least for accounting purposes, the intent is to record actual transactions that economic units and people have with the environment, i.e. the aim is to record past events. Recording these past events through an accounting framework is thus particularly important to underpin subsequent valuations.

References

1. Corine. CORINE Land Cover, http://land.copernicus.eu/pan-european/corine-land-cover. (2017).
2. ArcGIS. https://www.arcgis.com/home/item.html?id=c61ad8ab017d49e1a82f580ee1298931https://www.arcgis.com/home/item.html?id=c61ad8ab017d49e1a82f580ee1298931. (2016).
3. Dominati, E., Patterson, M. & Mackay, A. A framework for classifying and quantifying the natural capital and ecosystem services of soils. *Ecological Economics* **69**, 1858-1868, doi:10.1016/j.ecolecon.2010.05.002 (2010).
4. WAVES. *Wealth Accounting and the Valuation of Ecosystem Services. https://www.wavespartnership.org/*, 2017).
